# Patchiness of plankton ecosystem structure due to nutrient mixing along the shelf edge in the North Sea

**DOI:** 10.1038/s41598-024-83811-8

**Published:** 2025-01-07

**Authors:** Axelle Cordier, Jørgen Bendtsen, Niels Daugbjerg, Nikolaj From, Sigrún Huld Jónasdóttir, Erik Askov Mousing, Jens Tang Christensen, Teresa Silva, Katherine Richardson

**Affiliations:** 1 Globe Institute, Section for Biodiversity, University of Copenhagen, Universitetsparken 15, 2100 Copenhagen Ø, Denmark; 2 Globe Institute, Section for Geobiology, University of Copenhagen, Øster Voldgade 5-7, 1350 Copenhagen K, Denmark; 3 Marine Biological Section, Department of Biology, University of Copenhagen, Universitetsparken 4, 2100 Copenhagen Ø, Denmark; 4 National Institute of Aquatic Resources, Technical University of Denmark, 2800 Kgs. L1yngby, Denmark; 5 Department of Climate Modeling and Air Pollution, Norwegian Meteorological Institute, Postbox 43 Blindern, NO-0313 Oslo, Norway; 6 Department of Biology, Aarhus University, Ole Worms Allé 1, 8000 Aarhus C, Denmark; 7 Marine Pelagic Division, Marine and Freshwater Research Institute, Fornubudin 5, 220 Hafnafjordur, Iceland

**Keywords:** Biogeography, Ocean sciences

## Abstract

Mid-water column turbulence has been shown to cause elevated vertical nutrient flux at the shelf edge in the northeastern North Sea. Here, we demonstrate that phytoplankton communities in this region tend to be dominated by larger cells (estimated from percentage of chlorophyll captured on a 10 μm filter) than beyond the shelf edge. F_v_/F_m_ (PSII electron transport capacity) corrected for photoinhibition in the surface layer correlated in this study with the percentage of chlorophyll captured on a 10 µm filter (assumed to be large cells), suggesting that the phytoplankton community was responding to increased nutrients in the euphotic zone by increasing photosynthetic efficiency and altering community composition. The greatest abundances of larger copepods and the highest rates of *Centropages typicus* egg production were also generally found at the shelf edge. These results suggested that impact from increased nutrient fluxes cascaded up the planktonic food web. As these regions of nutrient flux were very localised, this led to sub-mesoscale heterogeneity in plankton ecosystem structure. Reports of higher abundances of fish and mammals at the shelf edge are common and we hypothesise that their distributions are a response to the impact of mid-water column nutrient upwelling on the plankton food web in the region.

## Introduction

An inherent characteristic of vertical and horizontal plankton distributions in marine and freshwater environments is their spatial patchiness^1,2^. This uneven distribution is often attributed to the interplay between physical (i.e., water movement), chemical (i.e., nutrient availability) and biological factors (i.e., predation)^3^. It is also often assumed that patchiness in the size distributions of plankton impacts the distribution of organisms populating higher trophic levels^4^. However, a mechanistic understanding of the processes driving the patchiness in the structure of plankton ecosystems is still lacking.

The role of turbulence in the stimulation of primary production in localised areas has been investigated and the potential for phytoplankton patches to influence the upper planktonic food web structures has been argued^5^. Furthermore, field studies frequently report elevated concentrations of zooplankton and fish larvae at marine fronts, leading to speculation that localised vertical mixing could be responsible for small-scale variations in plankton ecosystem structure compared to those found in waters distant from these frontal zones^6,7^. The specific plankton ecosystems at fronts are thus primary candidates for supporting the enhanced upper trophic levels associated with mixing areas^8^. We therefore expect that localised vertical mixing between nutrient-deplete and nutrient-rich water masses in the euphotic zone will be reflected in plankton food web structure.

The term *new production* (NP) was formulated^9^ to differentiate primary production supported by nitrogen coming from an external source from regenerated primary production, i.e., fuelled solely by nitrogen regenerated within the system. The f-ratio was later defined^10^ as the proportion of NP in the total primary production. When primary production is based on locally regenerated nutrients, production may be in an almost stable state and the f-ratio low, while input of nitrate into the euphotic zone can lead to a higher f-ratio and a net increase in phytoplankton biomass. Evidence from the permanently stratified Sargasso Sea indicated that where mid-water column vertical mixing occurs, there are hotspots of primary production (PP) with elevated f-ratios, and increased concentrations of eel larvae^11^. In contrast to the Sargasso Sea, the northeastern North Sea is only seasonally stratified. Low PP has traditionally been assumed to take place during the summer period when the water column is stratified. However, turbulence was measured at the shelf edge of the northeastern North Sea and localised vertical mixing was demonstrated^12^. A model study additionally showed the potential of this mixing to result in NP hotspots^13^. The region in which these hotspots were predicted to occur coincided with regions where high concentrations of fish larvae were reported^14^. Relatively high nitrate fluxes (> 0.5 mmol N m^−2^ d^−1^) were found to be caused by localised vertical mixing along the shelf edge^12^, bringing nutrient-rich deep waters into the euphotic zone. At the shelf edge, the nutricline was found to be significantly deeper^13^ and a breach in the stratified water column by localised mixing could explain the relatively high NP (~ 100 mg C m^−2^ day^−1^) estimates here. The region in which these localised events were identified is known to house a diverse and abundant fauna including top predators^15^.

A relatively short ‘traditional’ food web characterised by large phyto- and zooplankton can be differentiated^4^ from a relatively long one dominated by smaller organisms. It has been empirically shown that trophic transfer is more efficient when both prey and predator size diversity is higher^16^. The diversity and size structure of phytoplankton are thus believed to control how efficiently energy introduced to the food web via photosynthesis is passed on to higher trophic levels. Spawning fish populations are known to aggregate at shelf fronts^17^ (e.g. Celtic Sea), possibly due to enhanced food availability (i.e.., through an increase in mesozooplankton abundance), and are therefore more intensively fished there^18,19^. Therefore, in theory, productive areas characteristic of upwelling regions are associated with this specific ecosystem structure (phytoplankton communities dominated by large phytoplankton and relatively efficient transfer of energy through the planktonic food web) resulting in enhanced fish production^5,20,21^.

**Fig. 1:**
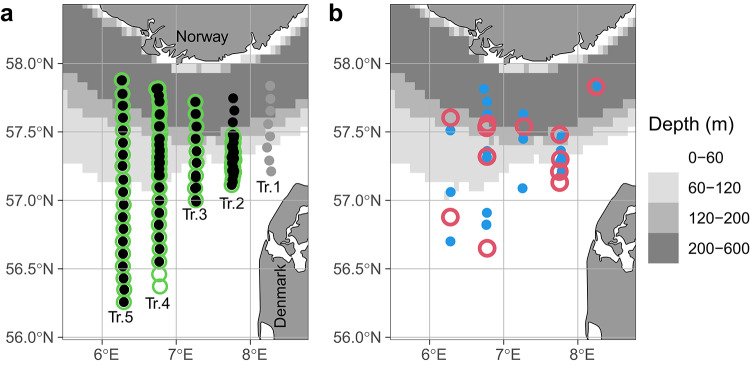
Position of samples collected at Transect 1–5 in the North Sea, (**a**) Water samples used for chlorophyll a measurement (solid black circles) and stations selected for F_v_/F_m_ measurements (green open circles), Transect 1 (solid grey circles) was excluded from the analyses. (**b**) Water samples used for egg production rates (blue solid circles) and zooplankton samples (pink open circles). Maps were produced using the ggOceanMaps package^23^ in R^24^.

Here, we empirically test the hypothesis that the impact of localised subsurface vertical mixing events cascades through the food web and is associated with spatial heterogeneity in plankton ecosystem structure. Using data collected along 5 transects (Fig. 1) on the VERMIX-cruise during July 2016, i.e., the same cruise used to demonstrate elevated vertical mixing in the shelf edge area^13^, we identify water patches in the same area as these mixing events with i) elevated variable fluorescence corrected for photoinhibition^22^, ii) elevated proportions of large phytoplankton and zooplankton, iii) increased abundance of zooplankton and iv) higher copepod egg production rate than in surrounding waters. These data validate the theory of a cascade effect, triggered by localised subsurface mixing of nutrients into the euphotic zone, up through the plankton food web. The localised mixing events appear to generate sub-mesoscale patchiness in the structure of the plankton ecosystems in the region of the shelf edge.

## Results

**Fig. 2:**
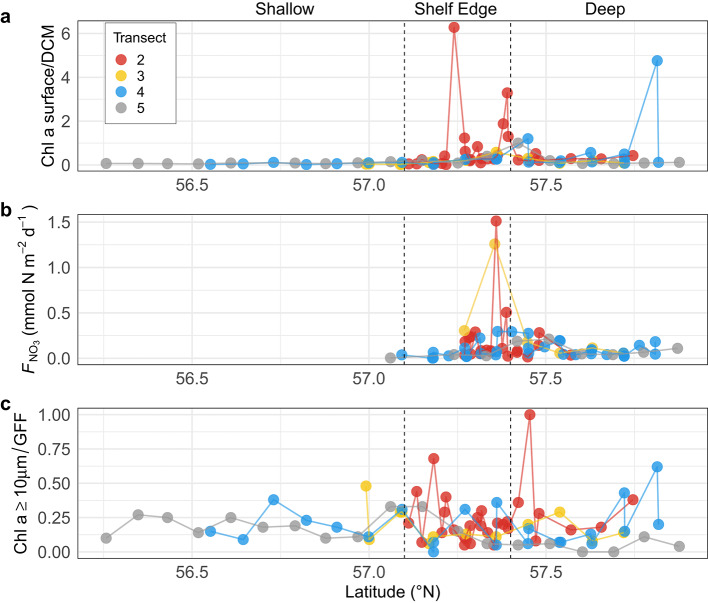
Measurements of chlorophyll *a* ratios and nitrate fluxes in shallow, shelf edge and deep areas. (**a**) Ratios of chlorophyll *a* concentration at surface layers (5 m) and DCM, (**b**) Maximum nitrate flux into the euphotic zone previously reported ^12^. Note that nitrate was not detectable above the shallow area, i.e., F_NO3_ = 0. (**c**) Surface (5 m) ratio of chlorophyll *a* ≥ 10 μm per GFF filter.

**Fig. 3:**
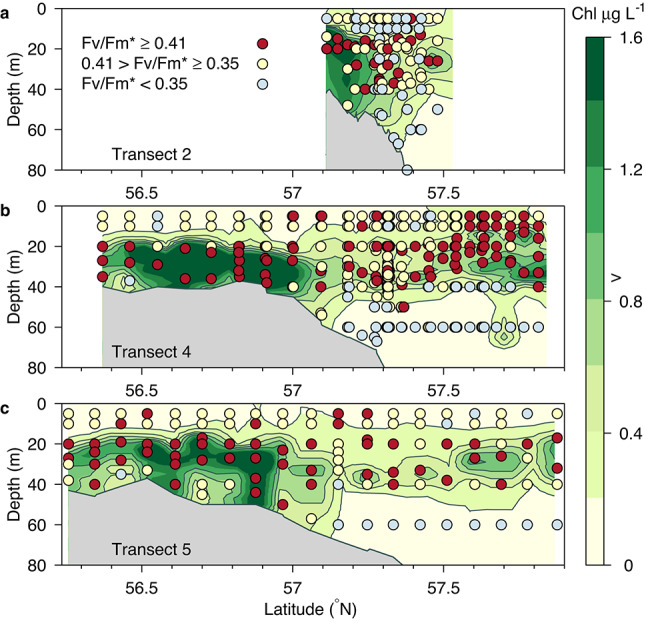
Distributions of F_v_/F_m_* below 0.35 (< mean—SD), within 0.35–0.41 (within mean ± SD) and above 0.41 (> mean + SD) at three different transects, (**a**) Transect 2, (**b**) Transect 4 and, (**c**) Transect 5. Chlorophyll *a* concentrations are shown as contours (Chlorophyll a, µg L^−1^). The figure was made using the scientific graphic language Gri (version 2.12.23, https://gri.sourceforge.net/).

**Fig. 4:**
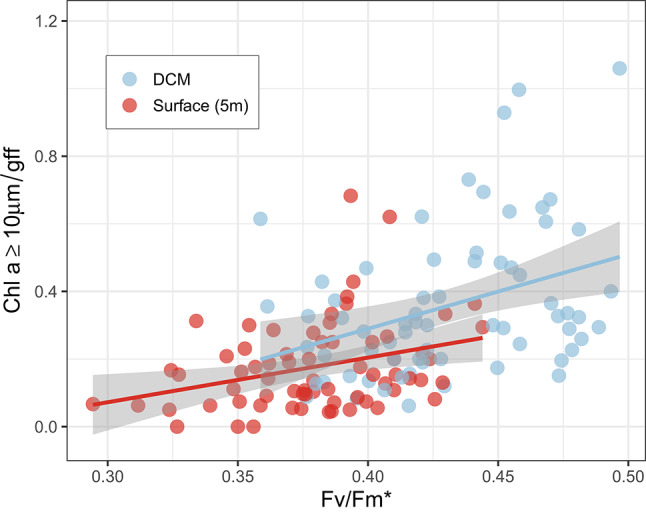
Relationship between Fv/Fm* and chlorophyll *a* ≥ 10 μm/GFF at the surface and DCM of all transects. Linear regressions were fitted to data from all transects, at the surface (R^2^ = 0.1, p < 0.01) and at the DCM (R^2^ = 0.16; p < 0.001). Shaded ribbon is the 95% confidence interval.

Primary and secondary producers were sampled in the northeastern North Sea (Fig. 1). As a basis of locating a signal of mixing, we additionally show nitrate flux estimates previously reported^12^. Analysis of the vertical distribution of chlorophyll *a* concentration showed a weakening of the deep chlorophyll maximum (DCM) at the shelf edge that spatially coincided with both elevated mixing, higher estimates of nitrate flux and greater heterogeneity in the proportion of large cells in the phytoplankton community (proportion of total chlorophyll *a* retained on a 10 μm filter (chlorophyll *a* ≥ 10 μm/GFF), with evident variations among transects in magnitude and spatial relation to nitrate fluxes (Fig. 2). F_v_/F_m_* was used as an indication of nutritional status of the phytoplankton community. Elevated F_v_/F_m_* in surface waters suggested that phytoplankton here had benefited from increased nutrient enrichment (Fig. 3). F_v_/F_m_* at the surface and at the DCM over the entire study area correlated significantly with chlorophyll *a* ≥ 10 μm/GFF (Fig. 4). A greater proportion of chlorophyll *a* ≥ 10 μm/GFF was assumed to correspond to a greater dominance of large cells in the phytoplankton community.

**Fig. 5:**
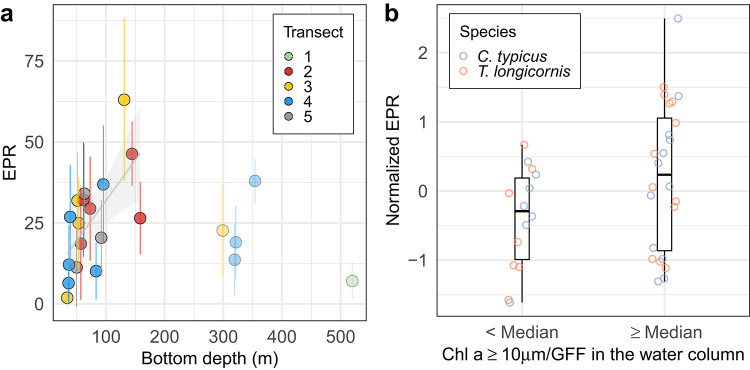
(**a**) Average (± standard error) of *C. typicus* egg production rate (EPR: eggs female^−1^ day^−1^) over bottom depth. A linear regression (R^2^ = 0.45, p < 0.05) was fitted on the data located at the shallow and shelf edge with 95% confidence interval (shaded ribbon), (**b**) Box plot of the normalised (Z-score) EPR for two copepod species (*C. typicus* and *T. longicornis*) when mean chlorophyll *a* ≥ 10 μm/GFF in the water column was below and above the median (= 0.28) of the entire data set. The box delimits the 25 and 75th percentile, and the whisker is the range. The comparison between the group’s mean was assessed with a t-test (p < 0.05).

**Fig. 6:**
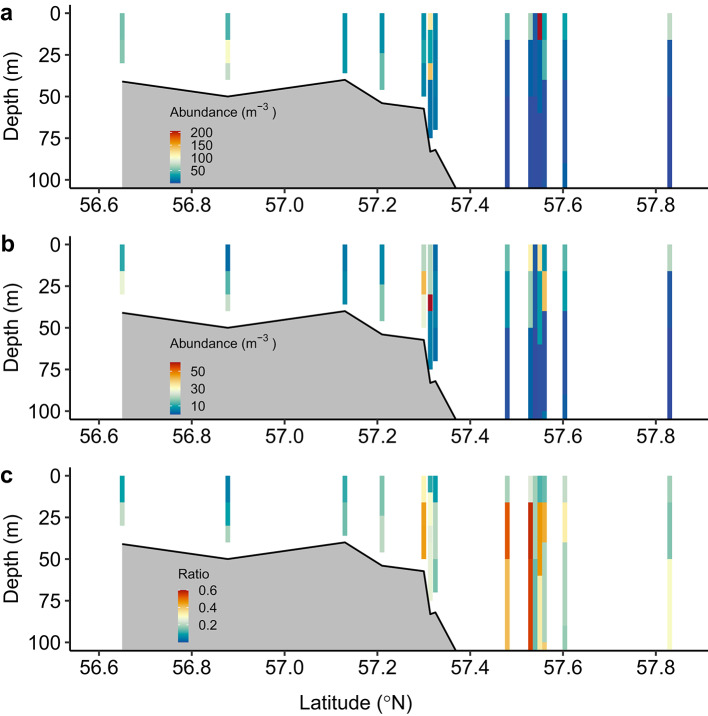
Mean values of vertical abundance of copepods over all transects, (**a**) size range 0.5–1 mm, (**b**) size range 1.5–2 mm and (**c**) the ratio of the 1.5–2 mm size range over total copepod abundances. The black line represents the bottom depth at the station sampled.

Egg production rates (EPR; eggs female^−1^ day^−1^) were determined for two copepods, *Centropages typicus* and *Temora longicornis*. EPR for *C. typicus* increased from shallow waters to the shelf edge region (Fig. 5a). The combined EPR data for both copepods were related to the predominance of larger phytoplankton (Fig. 5b) in the water column. Mapping of the distribution of “small” (0.5–1 mm) and “large” (1.5–2 mm) copepods indicated the greatest concentration of the larger copepods to be centered at the shelf edge (Fig. 6).

### DCM strength, nutrient fluxes and chlorophyll size fractions

While the southern and northern parts of the study area (Fig. 1), i.e. bottom depths < 60 m and deeper than 120 m, respectively, were generally characterised by the presence of a strong DCM, the chlorophyll *a* concentrations were much more heterogeneously vertically distributed at the intermediate depths along the shelf edge (Fig. 2a). The area in which the weakest DCM was observed coincided with the region (~57.25–57.50°N) in which the largest vertical nutrient fluxes were estimated (Fig. 2b). One station with a weakened DCM was also recorded at the northern end of Transect 4, where factors not associated with shelf-edge processes may be inducing mixing (see below). Thus, the vertical distribution of chlorophyll *a* in the study area appears to reflect the water column turbulence patterns and estimated vertical nutrient fluxes for the area^13^. Furthermore, the strongest signals of the weakened DCM were on Transect 2 and corresponded to nearby stations having the most elevated ratios of chlorophyll *a* ≥ 10 μm/GFF (Fig. 2c). In addition to the shelf edge, the station at the northern end of Transect 4 exhibiting a weakened DCM was associated with a greater dominance of large phytoplankton in the surface water community than elsewhere on this transect.

### Vertical mixing and surface nutrient status

F_v_/F_m_ represents electron transport efficiency in PSII and, when corrected for photoinhibition^18^, i.e., Fv/Fm*, it indicates the nutritional status of the phytoplankton community. For simplification, F_v_/F_m_* values were categorised into low (< mean—SD), medium (within mean ± SD) and high (> mean + SD) values. In general, F_v_/F_m_* values at the surface (≤ 10 m) were considerably lower than those recorded at the DCM (Fig. 3). This was consistent with the expected nutrient limitation of phytoplankton in the surface layer. Nevertheless, F_v_/F_m_* in the surface layer displayed patchiness (Fig. 3).

Relatively high surface F_v_/F_m_* values were found at some stations over the shelf edge, i.e., the region where vertical nutrient flux was estimated to be enhanced^12^. On Transect 2, the relatively high surface F_v_/F_m_* around 57.4°N matched well the strong signal of the DCM weakening, nitrate flux and high proportion of large cells in the phytoplankton community (Fig. 2). On Transect 4, relatively high surface values were also recorded at the northernmost stations of the transect, a region also associated with a weak DCM (Fig. 2a), and where the DCM was closer to the surface than in the remainder of the study area (Fig. 3). This northerly end of the transect was potentially influenced by Norwegian coastal waters, i.e., water outflow from the Baltic Sea^13^. On Transect 5, relatively high surface F_v_/F_m_* values were also recorded in the shallowest (southern) part of the transect (~ 40 m), where the shallow depth may explain a relatively larger mixing between surface and bottom layers due to wind and tidal forcing. Thus, the elevated F_v_/F_m_* in surface waters suggested that phytoplankton here had benefited from increased nutrient enrichment of the surface waters (Fig. 3).

### F_v_/F_m_* and phytoplankton size structure

The size structure of the phytoplankton communities (estimated from size fractionated chlorophyll determinations) along all transects at all depths was significantly related to F_v_/F_m_* (p < 0.01) with communities dominated by large cells having the highest F_v_/F_m_* (Fig. 4). Linking this result to F_v_/F_m_* distributions (Fig. 3), surface phytoplankton communities dominated by large cells were found at the shelf edge. The combined dataset included samples from the DCM, where communities tended to be more nutrient replete compared to surface waters. The inclusion of the DCM data increased the range of F_v_/F_m_* values included in the analysis. Note that ratios higher than 1 were seen from a few samples because filtering was made from independent replicates. Thus, in some cases, higher chlorophyll *a* was observed on larger filter size.

### Copepod egg production and zooplankton distribution

Egg production rates, i.e., EPR (eggs female^−1^ day^−1^), were determined for two copepods, *Centropages typicus* and *Temora longicornis.* We found a significant linear relationship (p < 0.01) describing an increasing trend in EPR for *C. typicus* from the shallow to the shelf edge area (Fig. 5a). Deep area samples were excluded from the analysis due to a lack of data points between bottom depths ~ 160-300 m. There, the few low values were consistent with the decreased turbulence activity north of the shelf edge, and the relatively high value on transect 4 (bottom depth ~ 353 m; latitude ~ 57.8°N) coincided with a zone exhibiting a weak DCM and high surface F_v_/F_m_* (Fig. 2). The highest mean EPR of *C. typicus* was found on Transect 3 in the water column with bottom depths ~ 127 m (Fig. 5a), corresponding to a peak in surface ratios of chlorophyll *a* ≥ 10 μm/GFF (latitude ~ 57.4°N; Fig. 2).

The EPR of both *C. typicus* and *T. longicornis* were normalised based on the mean and standard deviation: $\text{Z }= \left(\text{EPR}-\upmu \right) /\upsigma$, where μ is the mean of EPR of both species and σ its standard deviation. A t-test was conducted to assess the difference between the mean normalised EPR found at low (< Median) or relatively high (≥ Median) proportions of chlorophyll *a* ≥ 10 μm/GFF in the water column. The median of the chlorophyll *a* ≥ 10 μm/GFF in the water column was chosen as a threshold to categorise EPR values found in the lower and higher half of chlorophyll *a* ≥ 10 μm/GFF observations. We found EPR values to be significantly higher (p < 0.01) in the presence of relatively high proportions of large phytoplankton.

Communities dominated by smaller (0.5–1 mm) copepods were found over the entire study area, while communities dominated by the larger copepods (1.5–2 mm) were found only in the water column near the shelf edge at ~ 57.35°N and ~ 57.5°N (Fig. 6). Most of the copepod species identified (*Calanus*, *Oithona* and *Pseudocalanus*) followed the same pattern, except for *Temora* which seemed to be in lower abundance in the water column at the shelf edge compared to the shallow area (see Supplementary Fig. S2 online).

## Discussion

It has long been argued^4,5^ that nutrient enrichment by vertical mixing will lead to greater abundance of large phytoplankton and that this, in turn, will support the production and increase in abundance of mesozooplankton. Empirical demonstration of this cascade is, however, difficult partly because mixing events are often ephemeral but also owing to differences in sampling scales and time lags in responses of the different trophic levels. Directly linking phytoplankton and zooplankton community structure is hampered by the fact that the former is usually sampled at specific depths and the latter over the entire water column or in relatively large depth strata. In addition, water movements are continually transporting organisms during the time it takes for phytoplankton to respond to nutrient enrichment or for zooplankton to respond to changes in phytoplankton community structure. Thus, we may predict that responses in phyto- and zooplankton ecosystem structure to nutrient enrichment will be separated in time and space both from each other and from mixing events themselves.

Here, we take advantage of earlier work^12,13^ demonstrating that episodic vertical turbulent nutrient fluxes from deep to surface waters are more common over the shelf edge in the northeastern North Sea than in the regions to the north and south. Using data collected on the same cruise as these studies, we demonstrated greater heterogeneity in phytoplankton and zooplankton community characteristics in this region with areas with elevated vertical mixing. Furthermore, it was also in this region that we observed evidence of phytoplankton communities dominated by large cells to be present over the entire water column at some stations. These findings were consistent with the hypothesised^4,5^ relationships between nutrient mixing and plankton ecosystems.

That there may be a causal relationship between the mixing and the responses noted in the phytoplankton community was supported by the demonstration of a significant relationship between F_v_/F_m_* and the relative proportion of large cells in the community, both at the surface and at the DCM. The finding of a relationship between F_v_/F_m_* and the size structure of the phytoplankton community is particularly noteworthy as it may provide a more general tool for assessing relative differences in community size structure.

On land, it is well established that plant community composition is important both for food web structure and biological carbon sequestration. This seems likely to also be true for phytoplankton, but their small size makes the routine identification (i.e., based on light microscopy) of different plankton communities difficult. Using changes in size structure (established through size fractionated chlorophyll analysis) of phytoplankton communities as a proxy for changes in community diversity, it has been shown that differences in photosynthetic parameters^25^, responses to nutrient availability^26^, temperature^27^ and ocean-atmosphere carbon flux^28^ can all be related to community diversity. F_v_/F_m_* is to some degree dependent on species composition^29^ and does not, therefore, provide a quantitative indicator of community size structure. Nevertheless, if the general relationship noted here is found to be ubiquitous, then F_v_/F_m_* could potentially become a valuable tool for exploring differences in phytoplankton community distributions in nature.

Both the vertical chlorophyll distributions (Fig. 2) and the F_v_/F_m_* distributions (Fig. 3) found were consistent with the conclusion^12,13^ of vertical mixing occurring at the shelf edge. It seemed that the water column stability characteristics allowing the development of distinct DCMs north and south of the shelf edge were not present at the shelf edge. Elevated F_v_/F_m_* values suggest a relaxation of nutrient depletion in the ambient phytoplankton community, so the surface phytoplankton communities appeared to be more nutrient replete over the shelf edge than their counterparts to the north and south (although at the extremes of some of the transects other than shelf edge mixing processes appear also to influence surface F_v_/F_m_* and phytoplankton size structure, see Fig. 3).

Elevated F_v_/F_m_* was shown here to be associated with increasing dominance of large cells in the phytoplankton community (Fig. 4). Using F_v_/F_m_* as an indicator for community size structure, we conclude that communities dominated by larger photosynthetic organisms were found at the DCM and throughout the water column where vertical mixing is suggested to occur. Thus, we can predict greater retention (i.e., capture, ingestion) efficiency of the phytoplankton by the larger zooplankton grazers in these areas.

Copepod egg production depends on numerous factors, where food availability, type and quality are of importance^30^. It is believed that copepod egg production relates directly to food intake and that copepod retention of food particles greatly increases when particle size is > 10 μm^31^. We therefore suggest that enhanced EPR (Fig. 5) and predominance of large copepods (Fig. 6) at the shelf edge were related to a higher proportion of large phytoplankton. However, a time lag, and thus a spatial mismatch due to current activity^32^, between greater food retention and EPR may be expected. This may be reflected in the lack of linear relationship between EPR and percentage of large cells in the phytoplankton community (see Supplementary Fig. S3 online).

The overall findings of a response signal in planktonic communities to vertical mixing provide empirical evidence that physical characteristics of shelf fronts have the potential to provide food requirements necessary for fish larvae survival^14^ and thus fuel the production of higher trophic levels. Interestingly, pelagic fishing intensity in the Celtic Sea is concentrated at the edge of the continental shelf, which was suggested to be caused by internal tides^19^ that could in turn lead to NP. We therefore speculate that our results can explain the accumulation of organisms representing higher trophic levels in our study area^15^.

## Conclusion

This study demonstrates sub-mesoscale differences in phytoplankton community size structure that appear to influence both zooplankton distributions and egg production. Geographically, these differences in phytoplankton community structure were associated with a region where episodic upwelling of nutrient-rich deep water to the euphotic zone is known to exist. A causal link between this upwelling and the differences noted in community size structure was suggested by the significant positive relationship between F_v_/F_m_* and dominance of larger phytoplankton in the community, as higher values indicate a relaxation of nutrient depletion. The increased copepod egg production and dominance of larger cells in the phytoplankton community at the shelf edge further suggested a signal propagating from the localised upwelling to higher trophic levels. We suggest that such upwelling-induced sub-mesoscale differences in the structure of plankton ecosystems may be an underlying driver of “patchiness” in the distribution of marine organisms at even higher trophic levels^15,19,33^.

## Methods

### Study area

The VERMIX cruise (12–31 July 2016) in the northeastern North Sea sampled along five transects (Tr.1–5) and F_v_/F_m_ measurements were taken on Tr. 2–5 and were used for the phytoplankton analyses. Measurements of turbulence, hydrography, nutrients and chlorophyll *a* distributions and sampling conditions were described in a previous study^12^. Measurements of nitrate and phosphate^12^ suggest that denitrification might be occurring in the shallow area (< ~ 60 m) south of the shelf edge towards the central North Sea. More recently, the shallow and the shelf edge area were shown to be weakly connected whereas a larger connectivity was found between the shelf edge and water above the Norwegian trench^28^. Therefore, we refer to visited stations in three groups: *shallow* (< 60 m), *shelf edge* (60–120 m), and *deep* (> 120 m), as defined in a previous study^13^.

### F_v_/F_m_ corrected for photoinhibition (F_v_/F_m_*)

At each station, water was collected from 4 selected depths for determination of chlorophyll *a* fluorescence kinetics, transferred directly to a 300 ml dark glass flask and stored for ~ 30 min until measurement of F_v_/F_m_. Subsequently, 100 ml of sample were transferred to a 200 ml transparent glass flask and incubated for ~ 4 h at 50 μmol photons m^-2^ s^-1^ in a temperature-controlled room, with a temperature adjusted to match the average temperature of the collected water samples. This light treatment has been shown to remove any signal in the F_v_/F_m_ caused by photoinhibition^18^. After the incubation, F_v_/F_m_ was again determined. Differences in post-incubation F_v_/F_m_ were assumed to reflect differences in nutrient status in the phytoplankton communities with low F_v_/F_m_* values indicating nutrient stress.

F_v_/F_m_ was measured using a FastOcean FRRf 3 sensor (Chelsea Technologies Group, UK) with a dark chamber installed, and data was acquired in the FASTpro 8 software (Chelsea Technologies Group, UK). A single turnover protocol was used with a saturation phase consisting of 100 flashlets with a 2 μs pitch and 16 sequence repetitions. For each measurement, the sensitivity (PMT eht) was automatically optimised by the software and the LED light intensity (E_LED_) was manually optimised. The saturating light was composed of 450, 530, and 624 nm wavelengths in a constant ratio of 1:0.5:0.8. The detection limit of the system was F_v_/F_m_ = 0.15. All values below the detection limit were presented as 0.15 here. For depths < 30 m, the highest F_v_/F_m_ value (dark/light incubated) was presented to reflect the maximum PSII electron transport potential. For depths > 30 m, the F_v_/F_m_ as a result of dark incubation only was presented as no photoinhibition was expected at these depths.

### Chlorophyll

Total chlorophyll *a* and size fractions of chlorophyll *a* were determined at selected stations at 5 m, the depth of the deep chlorophyll *a* maximum (DCM) and at 30 m. Seawater samples tapped from Niskin bottles and filtered through Whatman GFF (0.7), 3, and 10 μm pore size Millipore filters. For each sampling depth and filter size, triplicate filtrations of 200 − 500 ml seawater for the GFF filters and 400 − 1000 ml seawater for the 3 and 10 μm filters were performed. The samples were immediately frozen and later extracted for a minimum of 6 h in 5 ml ethanol (96%) in the dark at 6 °C. Chlorophyll *a* concentrations were measured using a Triology Laboratory Fluorometer (Turner Designs, CA, USA), which had been calibrated using a chlorophyll *a* standard from the DHI Group (Hørsholm, Denmark).

### Copepod egg production rate (EPR)

*Centropages typicus* and *Temora longicornis* females were collected at 23 stations with a 180 µm mesh plankton net, (1 m, ring diameter) fitted with a 5 L non-filtering cod-end. The sampling depth was about 2 m under the DCM and the net was towed vertically with a towing speed of approximately 15 m per minute.

The contents of the cod-end were gently transferred into a bucket with surface (5 m) water from the respective stations. Immediately after sampling, 20 active undamaged females were selected under a stereomicroscope and introduced into 250 ml bottles (1–3 females per bottle) filled with 64 µm screened ambient water. Four to 11 replicates were incubated per station. Screening was done in order to remove all eggs and nauplii from the initial incubation water. Females were incubated in darkness for 24 h at temperatures appropriate for the ambient temperature at the station where they were collected. The incubation was terminated by filtering the content of the bottles onto a 20 µm sieve and then washing the sieve contents into a small Petri-dish. Females were then removed and measured for length and the eggs counted.

### Zooplankton sampling and processing

Zooplankton samples were collected at 14 stations (latitudes ranging between 56.65–57.83°N and longitudes between 6.28–8.25°E) (See Supplementary Table S1 online) using a Multinet (Hydro-Bios, Kiel; mesh size 200 µm). Zooplankton were stored in 4% formalin in the freezer. Samples were size fractionated using a sieve and zooplankton split into two size groups, > 640 µm and < 640 µm. Samples were then digitally scanned on an Epson Perfection V800 photo (2400 dpi, 16 bit gray), using the software Vuescan (version 9.5, https://www.hamrick.com/). In total, 1340 images were produced, representing 134 samples (5 images for each sub-sample). Images were processed and statistics with the zooimage package^34^ and after the creation of a classifier, individuals were automatically identified, counted.

Raw scanned images were processed using ImageJ software (version 1.52, https://imagej.net/ij/) through the zooimage plugin “Scanner_Gray16”. Sub-images called vignettes were produced, representing every organism or particle detected by the plugin. Given the large diversity of zooplankton, a reasonable trade-off between taxonomy identification and resolution was needed to reduce errors^35^. The training set was composed of ten representative samples randomly chosen (different depths and transects) to be classified manually. Vignettes were grouped per class through the Xnview software (version 2.30). The classifier was assessed by the calculation of a tenfold cross-validation between the manual and automated classifications (Fig. S1). Additional tests were completed through an analysis of the precision and recall F-score ratios, and a manual validation of the classifier. The final classifier had a global error of 7.2% (F = 0.91).

Abundance per size (Equivalent Circular Diameter (ECD)) spectra (< 0.5, 0.5–1, 1–1.5, 1.5–2 and 2–2.5 mm) was obtain on copepods taxa. The size spectra 0.5-1 mm and 1.5–2 mm were consistently the most abundant groups in the samples. Therefore, subsequent analyses were conducted on these two size spectra which were assumed to be representative of the copepod communities in the area.

## Supplementary Information

Supplementary Information 1.

Supplementary Information 2.

Supplementary Information 3.

Supplementary Information 4.

Supplementary Information 5.

Supplementary Information 6.

## Data Availability

The datasets generated during the study are available in the supplementary information files.
