# Supplementary material for: Patchiness of plankton ecosystem structure due to nutrient mixing along the shelf edge in the North Sea
Source: Sci Rep. 2025 Jan 7;15:1183. doi: 10.1038/s41598-024-83811-8 (PMC11707148; doi:10.1038/s41598-024-83811-8)
Supplement: Supplementary file 6 — Supplementary Information 6. [file 41598_2024_83811_MOESM6_ESM.pdf]

Supplementary information

Patchiness of plankton ecosystem structure due to nutrient mixing along the shelf edge in the North Sea

Axelle Cordier[1*], Jørgen Bendtsen[2], Niels Daugbjerg[3], Nikolaj From[1], Sigrún Huld Jónasdóttir[4], Erik Askov Mousing[5], Jens Tang Christensen[6], Teresa Silva[7], Katherine Richardson[1]

[1] Globe Institute, Section for Biodiversity, University of Copenhagen, Universitetsparken 15, 2100 Copenhagen Ø, Denmark.

[2] Globe Institute, Section for Geobiology, University of Copenhagen, Øster Voldgade 5-7, 1350 Copenhagen K, Denmark.

[3] Marine Biological Section, Department of Biology, University of Copenhagen, Universitetsparken 4, 2100 Copenhagen Ø, Denmark.

[4] National Institute of Aquatic Resources, Technical University of Denmark, DK-2800 Kgs. Lyngby, Denmark

[5] Norwegian Meteorological Institute, Department of Climate Modeling and Air Pollution, Postbox 43 Blindern, NO-0313 Oslo, Norway

[6] Department of Biology, Aarhus University, Ole Worms Allé 1, DK-8000 Aarhus C, Denmark

[7] Marine Pelagic Division, Marine and Freshwater Research Institute, Fornubudin 5, 220 Hafnafjordur, Iceland

*E-mail: axelle.cordier@sund.ku.dk

Table S1. Stations selected for zooplankton sampling. Coordinates and sampling information of zooplankton samples.

Transect	Station	Latitude (°N)	Longitude (°E)	Bottom depth (m)	Replicates	Start depth (m)	Stop depth (m)
1	11	57.83	8.25	516	3	16	0
1	11	57.83	8.25	516	2	50	16
1	11	57.83	8.25	516	3	200	50
1	11	57.83	8.25	516	3	300	200
1	11	57.83	8.25	516	3	500	300
2	24	57.21	7.75	54	3	24	0
2	24	57.21	7.75	54	3	46	24
2	35	57.48	7.76	163	3	16	0
2	35	57.48	7.76	163	3	50	16
2	35	57.48	7.76	163	3	150	50
2	42	57.13	7.75	40	3	16	0
2	42	57.13	7.75	40	3	36	16
2	62	57.30	7.76	57	6	16	0
2	62	57.30	7.76	57	5	30	16
2	62	57.30	7.76	57	5	50	30
3	49	57.54	7.27	245	2	50	0
3	49	57.54	7.27	245	2	170	50
3	49	57.54	7.27	245	2	238	170
4	55	57.54	6.77	172	3	16	0
4	55	57.54	6.77	172	3	60	16
4	55	57.54	6.77	172	3	170	60
4	80	57.53	6.77	172	3	16	0
4	80	57.53	6.77	172	3	50	16
4	80	57.53	6.77	172	2	140	50
4	108	56.65	6.78	41	3	16	0
4	108	56.65	6.78	41	3	30	16
4	122	57.56	6.78	174	3	16	0
4	122	57.56	6.78	174	3	40	16
4	122	57.56	6.78	174	3	100	40
4	122	57.56	6.78	174	3	120	100
4	122	57.56	6.78	174	3	150	120
4	128	57.32	7.29	80	3	16	0
4	128	57.32	7.29	80	3	50	16
4	128	57.32	7.29	80	3	70	50
4	130	57.32	6.77	83	3	10	0
4	130	57.32	6.77	83	3	30	10
4	130	57.32	6.77	83	3	40	30
4	130	57.32	6.77	83	3	75	40
5	88	57.60	6.28	154	3	16	0
5	88	57.60	6.28	154	3	40	16
5	88	57.60	6.28	154	3	90	40
5	88	57.60	6.28	154	3	140	90
5	97	56.88	6.28	50	3	16	0
5	97	56.88	6.28	50	3	30	16
5	97	56.88	6.28	50	3	40	30

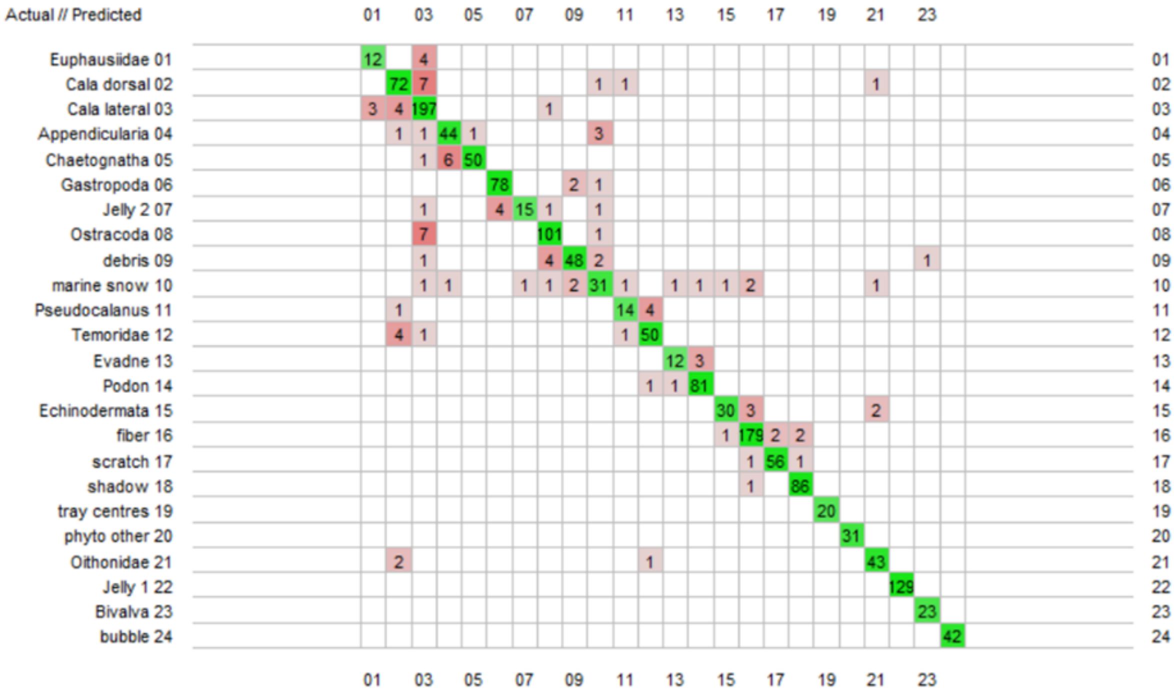

Figure S1. Zooplankton classifier assessment. Confusion matrix of the final classifier tested with the random forest algorithm. Rows are groups classified in the training set and columns are groups classified by the classifier. The green diagonal represents the number of vignettes the programme correctly classified. Boxes coloured in red are the wrongly classified organisms (light to darker red represents low to higher proportions).

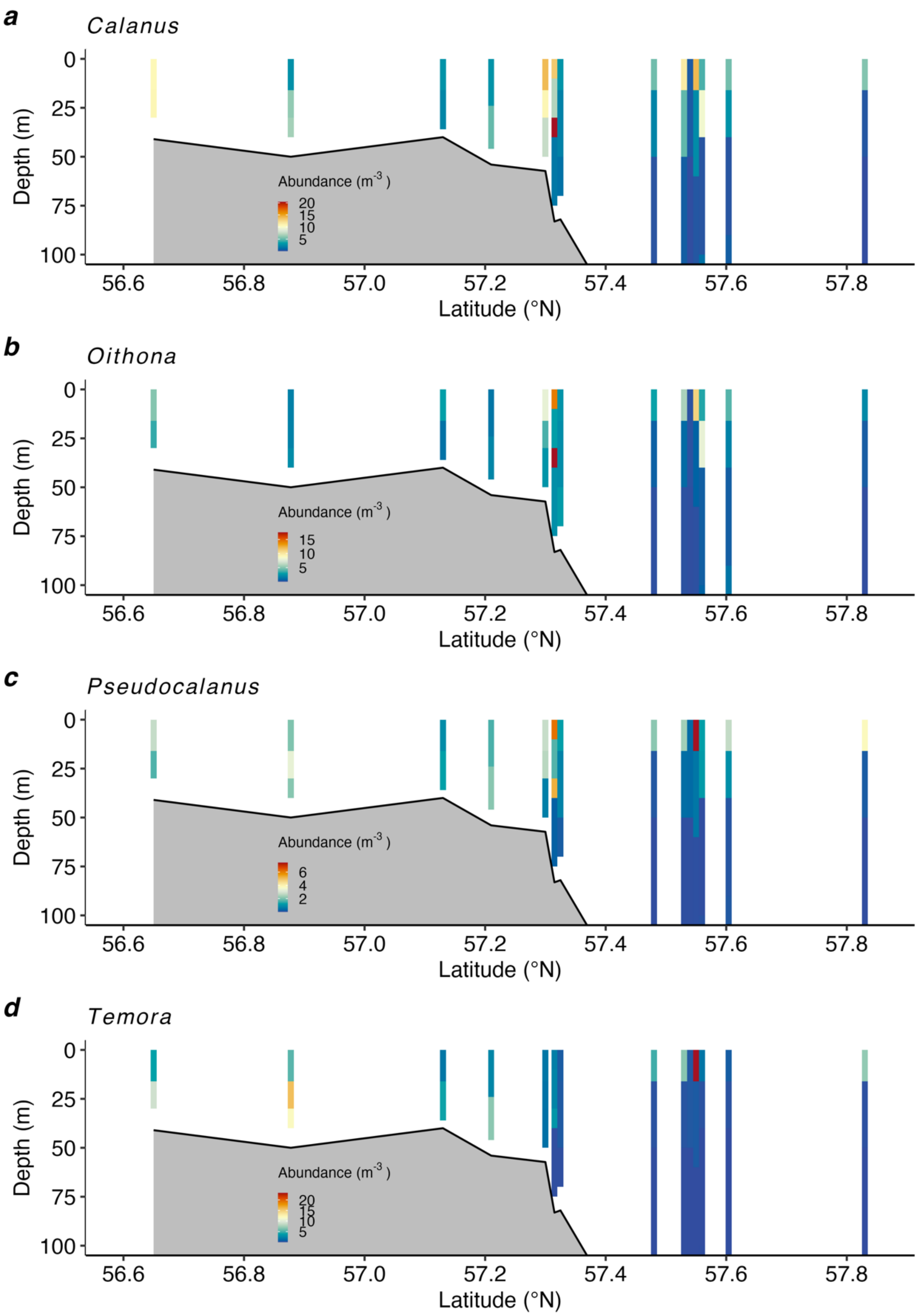

Figure S2. Distribution of copepod species. Mean abundance (number m^{-3}) of (a) *Calanus*, (b) *Oithona*, (c) *Pseudocalanus* and (d) *Temora* (all sizes considered) over all transect. The black line represents the bottom depth at the station sampled.

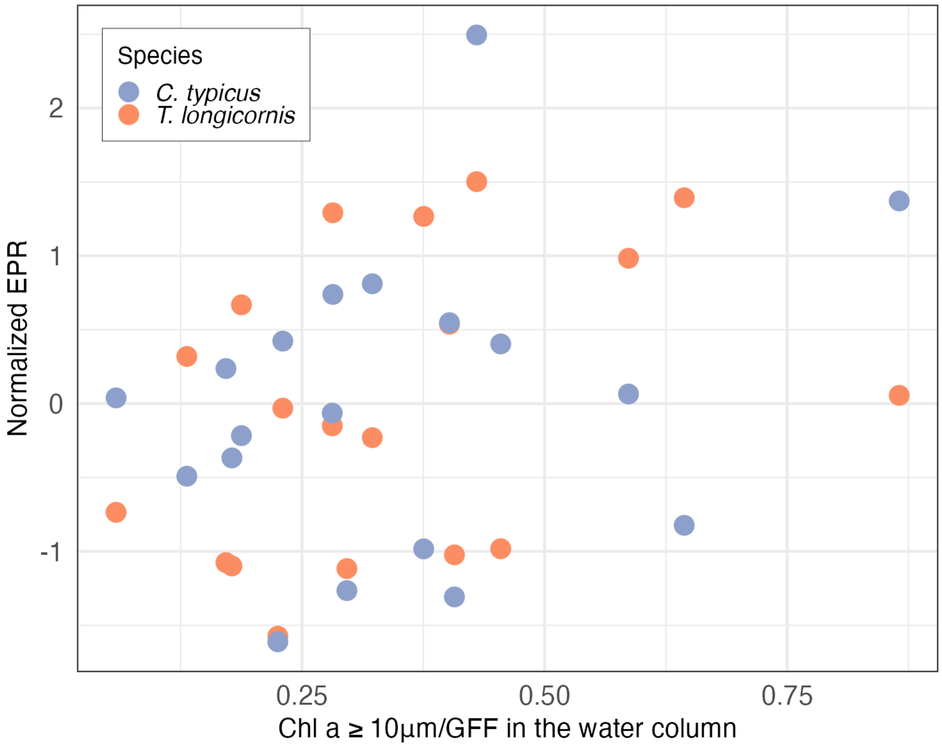

Figure S3. EPR and chlorophyll a ≥ 10 µm/GFF. Normalised (Z-score) EPR (eggs female^{-1} day^{-1}) for two copepod species (*C. typicus* and *T. longicornis*) against mean chlorophyll *a* ≥ 10 µm/GFF in the water column.

References

[1] Philippe, G. & Kevin, D. Supervised classification of images, applied to plankton samples using R and Zooimage. *Data Mining Applications with R.* 331–365; https://doi.org/10.1016/B978-0-12-411511-8.00013-X (2014).

[2] Gislason, A. & Silva, T. Comparison between automated analysis of zooplankton using ZooImage and traditional methodology. *J. Plankton Res.* **31**, 1505–1516; https://doi.org/10.1093/plankt/fbp094 (2009).
